# miRNA-Gene Interaction Network Construction Strategy to Discern Promising Traditional Chinese Medicine against Osteoporosis

**DOI:** 10.1155/2022/9093614

**Published:** 2022-06-15

**Authors:** Lubing Li, Xiahatai Ayiding, Ran Han

**Affiliations:** ^1^Department of Orthopedics, Chengdu Seventh People's Hospital (Tianfu District), Sichuan 610299, China; ^2^Department of Orthopedics, The Sixth Affiliated Hospital of Xinjiang Medical University, Xinjiang 830002, China; ^3^Department of International Ward, The Sixth Affiliated Hospital of Xinjiang Medical University, No.39 Wuxingnanlu, Urumqi, Xinjiang 830002, China

## Abstract

Osteoporosis is a widespread bone disease that affects million cases annually. The underlying mechanisms behind the progress of osteoporosis remain enigmatic, which limits detections of biomarkers and therapeutic targets. Hence, this study was aimed at exploring hub molecules to better understand the mechanism of osteoporosis development and discover the traditional Chinese medicine potential drugs for osteoporosis. miRNA and gene expression profiles were downloaded from Gene Expression Omnibus (GEO). Weighted correlation network analysis (WGCNA) was used to identify the key modules for osteoporosis. DIANA Tools was applied to perform pathway enrichment. A miRNA-gene interaction network was constructed, and hub miRNAs and genes were distinguished using Cytoscape software. Receiver operating characteristic (ROC) curves of hub miRNAs and genes were plotted, and correlations with hub genes and osteoporosis-associated factors were evaluated. Potential drugs for osteoporosis in Traditional Chinese Medicine Systems Pharmacology Database and Analysis Platform (TCMSP) were screened, and molecular docking models between these drugs and target genes were showed by AutoDock tools. Two hub modules, 1 miRNA module and 1 gene module, were identified to be the most strongly correlated with osteoporosis by using WGCNA. Then, 3 KEGG pathways including focal adhesion, PI3K-Akt signaling pathway, and gap junction were shared pathways enriched with the miRNAs and genes screened out by WGCNA and differential expression analyses. Finally, after constructing a miRNA-gene interaction network, 6 hub miRNAs (hsa-miR-18b-3p, hsa-miR-361-3p, hsa-miR-484, hsa-miR-519e-5p, hsa-miR-940, and hsa-miR-1275) and 6 hub genes (THBS1, IFNAR2, ARHGAP5, TUBB2B, FLNC, and NTF3) were detected. ROC curves showed good performances of miRNAs and genes for osteoporosis. Correlations with hub genes and osteoporosis-associated factors suggested implicational roles of them for osteoporosis. Based on these hub genes, 3 natural compounds (kainic acid, uridine, and quercetin), which were the active ingredients of 192 herbs, were screened out, and a target-compound-herb network was extracted using TCMSP. Molecular docking models of kainic acid-NTF3, uridine-IFNAR2, and quercetin-THBS1 were exhibited with AutoDock tools. Our study sheds light on the pathogenesis of osteoporosis and provides promising therapeutic targets and traditional Chinese medicine drugs for osteoporosis.

## 1. Introduction

Osteoporosis is the most common bone disease with clinicopathological features of low bone mineral density (BMD) and deterioration of bone microstructure [1]. Osteoporosis affects up to 40% women, and more than 3 million cases in US suffer from osteoporosis per year [2]. Bone is a living tissue in which the bone formation by osteoblast and bone resorption by osteoclast are in critical and dynamic balance [1]. Despite the universality of osteoporosis, the underlying mechanisms related to its progress are still ambiguous, which severely limits detections of biomarkers and therapeutic targets.

MicroRNAs (miRNAs) are short noncoding RNAs able to regulate gene expression by interacting with the 3′ untranslated region of target mRNA. Multiple studies have indicated that various miRNAs are involved in the process of bone metabolism and remodeling. Numerous miRNAs are dysregulated in patients with osteoporosis, including miR-194, miR-16, miR-338, miR-2861, miR-223, miR-422, miR-133, miR-21, miR-186, and miR-3651 [3–8]. For example, miR-194 was upregulated in osteoporosis patients and may be a promising biomarker for osteoporosis [4]. In addition, miR-16 was suggested to inhibit osteogenesis by suppressing the expression of VEGFA [5]. Moreover, miRNAs could regulate several signaling pathways involved in osteoporosis. For instance, miR-133 and miR-203 promote osteogenic differentiation [9, 10]. However, there is no study comprehensively analyzing hub miRNAs. In this study, we aimed to screen key miRNAs and their target genes by using comprehensive bioinformatics analysis.

Currently, high-throughput sequencing and bioinformatics technology has dramatically improved clinical, biological, and drug research. Weighted correlation network analysis (WGCNA) was a widely used computational tool for identification of hub genes [11]. In the present study, we constructed a coexpressed network with WGCNA and identified novel hub miRNAs and genes associated with osteoporosis. It is well established that traditional Chinese herbal medicine (TCHM) has been widely applied for thousands of years to treat diverse diseases, including osteoporosis [12]. However, multiple TCHMs have been reported to exert favourable antiosteoporosis actions through combining with Western medicine [13]. Herewith, it would be ponderable to distinguish the effective TCHM compounds used for treating osteoporosis. Based on it, this study established the target-compound-herb network to identify the significant target-compound.

## 2. Materials and Methods

### 2.1. Data Selection

miRNA profiles in GSE74209 and GSE93883 datasets were downloaded from Gene Expression Omnibus (GEO). Gene expression data were from GSE7158 and GSE35956 datasets. GSE74209 dataset contained 6 osteoporosis and 6 healthy cases. GSE93883 dataset contained 12 osteoporosis and 6 healthy cases. GSE7158 dataset contained 12 osteoporosis and 14 healthy cases. GSE35956 dataset contained 5 osteoporosis and 5 healthy cases. miRNA profiles from GSE74209 and GSE93883 datasets and mRNA profiles from GSE7158 and GSE35956 datasets were, respectively, merged and normalized using “normalization” package in R. Differentially expressed miRNAs (DEM) and genes (DEGs) were identified using “limma” package in R.

### 2.2. Weighted Correlation Network Analysis (WGCNA)

After detecting the outliers, “wgcna” package in R was applied to construct a WGCNA coexpression network. Firstly, soft thresholding power *β* was selected to ensure scale-free distribution (sale free *R*^2^ = 0.9). Secondly, the coexpression network was constructed based on topological overlap matrix (TOM). And the cluster dendrogram and the module colors were plotted to display the modules. For miRNA, the dissimilarity degree of module Eigengenes (MEDissThres) was set to 0.25. And the minModuleSize was set to 30. Modules with these parameters were merged into a new module. Pearson's correlation coefficients were analyzed to exhibit the correlation between clinical traits and the module. *P* < 0.01 was seen as significant.

### 2.3. Functional Enrichment Analysis

DEMs were subjected to DIANA Tools (http://www.microrna.gr/) to perform KEGG pathway analysis. “KEGG” package in R was used to enrich DGE-related KEGG pathways. The signaling pathways were seen to be significant when *P* < 0.05.

### 2.4. miRNA-mRNA Network Analysis

To examine the relationship between DEMs and DEGs, “network” package in R was downloaded. A miRNA-gene interaction network was constructed using Cytoscape software. The target genes of miRNAs were predicted by miRWalk.

### 2.5. Receiver Operating Characteristic (ROC) Analysis for Key miRNAs and Genes and Correlation with Key Genes and Crucial Osteoporosis-Related Genes

ROC analysis was conducted to assess the predictive performance of hub miRNAs and genes, and the predictive values of the area under the curves (AUCs) of ROC curves specific to hub miRNAs and genes were calculated. In addition, correlations with these key genes and vital osteoporosis-associated factors were calculated to evaluate functional roles of these genes in osteoporosis.

### 2.6. Development of Molecule Docking Model

The 2D structures of chemicals were downloaded from PubChem database. ChemBio3D Ultra 14.0 software was applied to draw the 3D structures of the chemicals. The structure with minimum free energy conformation was saved as a mol2 file. The protein of gene was searched in UniPort database, and the 3D structure of the target protein was downloaded from the Protein Data Bank (PDB) archive and transformed into 2D structure with PyMOL software and saved as a PDB file. Then, the molecular docking model was developed using the AutoDock Vina software. The model with the minimum free energy conformation was visualized with PyMOL software.

## 3. Results

### 3.1. Identification of Osteoporosis-Associated miRNAs

**Figure 1:**
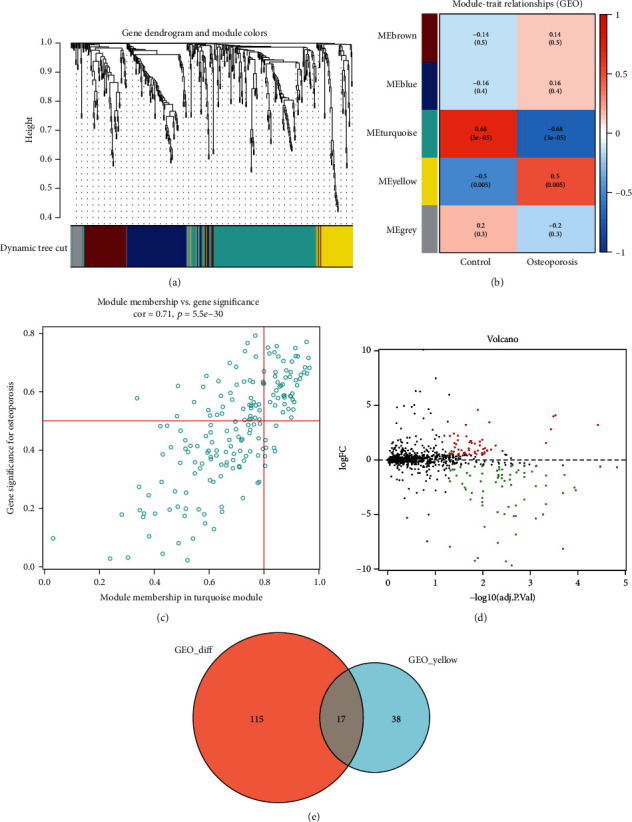
Identification of osteoporosis-associated miRNAs. (a) Hierarchical clustering tree and the module colors were plotted to display the modules. (b) The correlations between the 5 modules and osteoporosis. (c) The correlation between module membership and gene significance in the turquoise module. (d) The differentially expressed miRNAs were screened out in 18 osteoporosis and 12 healthy cases with the cutoff criteria adjusted *P* value < 0.01 and |log2FC| ≥ 1. (e) Venn diagram displayed the shared miRNAs between 132 differentially expressed miRNAs and 55 miRNAs in the turquoise module.

After combination and normalization of miRNAs from GSE74209 and GSE93883 datasets, 1,810 miRNAs in 18 osteoporosis and 12 healthy cases were obtained (Table 1). To screen out miRNAs correlated with osteoporosis, WGCNA was performed to construct a miRNA coexpression network. No outliers were detected in the 30 samples (Supplementary Figure 1A). The soft thresholding power *β* was selected as 12 for scale-free network (scale-free *R*^2^ = 0.9 with relatively high mean connectivity) (Supplementary Figure 1B). Then, 5 modules were obtained after similar modules were merged with the settings MEDissThres = 0.25 and minModuleSize = 30 (Figure 1(a)). The correlation between the 5 modules and osteoporosis was shown in Figure 1(b), and the strongest negative correlation was observed between the turquoise module and osteoporosis (*R* = −0.68, *P* = 3e − 05).  Figure 1(c) displays high correlation between module membership and gene significance in the turquoise module, suggesting that the miRNAs in this module were strongly associated with osteoporosis. Therefore, further analyses were performed with 55 miRNAs in turquoise module.

Further, to identify osteoporosis-associated miRNAs, we screened out the DEMs in 18 osteoporosis and 12 healthy cases. 132 DEMs including 56 upregulated and 76 downregulated miRNAs were identified in response to osteoporosis with the cutoff criteria adjusted *P* value < 0.01 and |log2FC| ≥ 1 (Figure 1(d)). The overlapped genes between 132 DEMs and 55 miRNAs in the turquoise module were selected as miRNAs associated with osteoporosis (Figure 1(e)), and 17 miRNAs were identified.

### 3.2. Identification of Osteoporosis-Associated Genes

**Figure 2:**
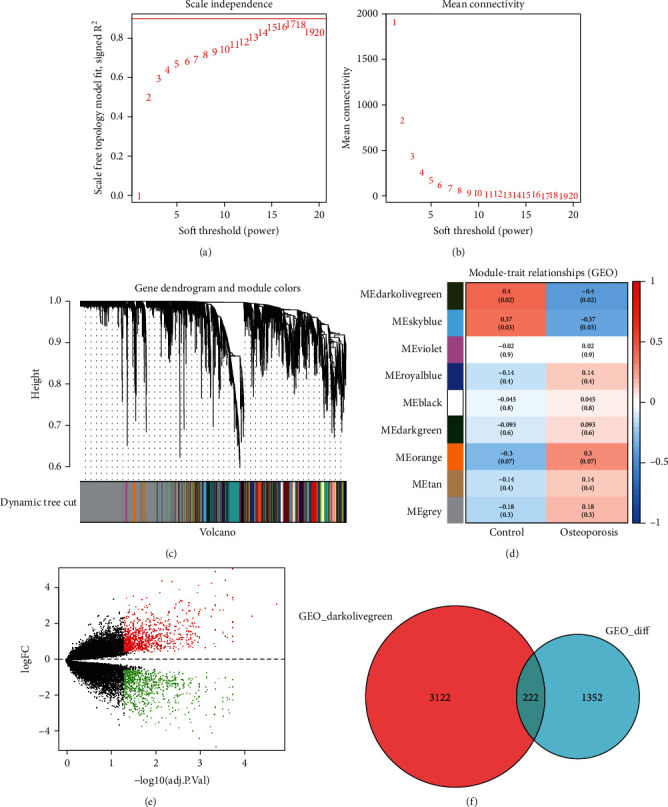
Identification of osteoporosis-associated mRNAs. (a) The soft thresholding power *β* was selected as 15 (sale free *R*^2^ = 0.9) to ensure a scale-free network. (b) Hierarchical clustering tree and the module colors were plotted to display the modules. (c) Nine modules were obtained with Pearson's correlation coefficients and *P* values. (d) The differentially expressed genes were screened out in 17 osteoporosis and 19 healthy samples with the cutoff criteria adjusted *P* value < 0.01 and |log2FC| ≥ 1. (e) Venn diagram displayed the shared miRNAs between 1,572 differentially expressed genes and 3,344 genes in the turquoise module.

In GSE7158 and GSE35956 dataset, 23,520 mRNAs were identified in 17 osteoporosis and 19 healthy samples (Table 2). The 36 samples were then subjected to WGCNA analysis. The soft thresholding power *β* was selected as 15, and 9 modules were obtained (Figures 2(a)–2(c)). The darkolivegreen module contained 3,344 mRNAs that were the most strongly correlated with osteoporosis (Figure 2(c)). 1,572 DEGs including 807 upregulated and 765 downregulated genes were identified in response to osteoporosis (Figure 2(d)). The 222 osteoporosis-associated shared genes were obtained after comparing the 3,344 genes and 1,572 DEGs (Figure 2(e)).

### 3.3. KEGG Pathway Analysis and Construction of miRNA-Gene Interaction Network

**Figure 3:**
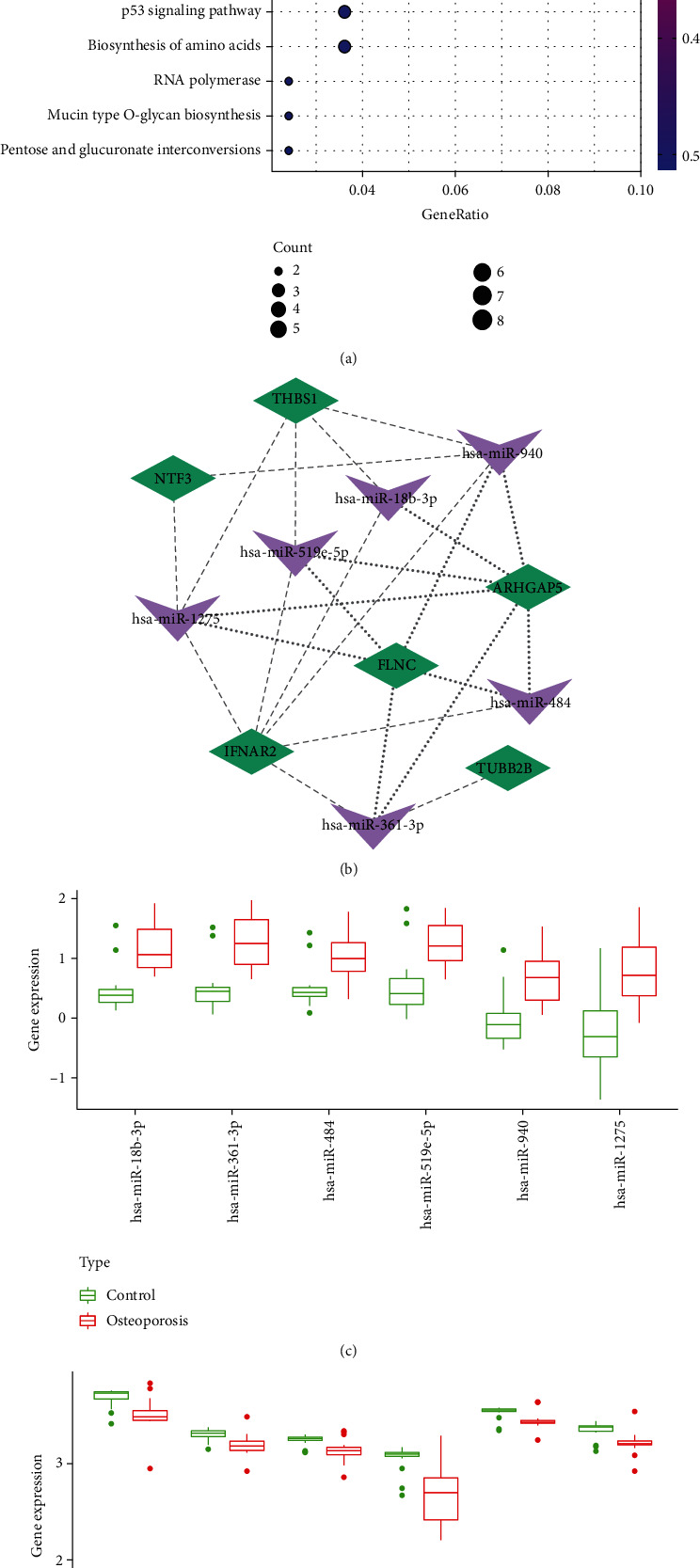
KEGG pathway analysis and construction of miRNA-gene interaction network. (a) Fourteen KEGG pathways were significantly enriched with the 222 osteoporosis-associated genes. (b) The miRNA-gene interaction network was constructed with Cytoscape software. (c) The expression of 6 hub miRNAs in 18 osteoporosis and 12 healthy cases. (d) The expression of 6 hub genes in 17 osteoporosis and 19 healthy samples.

Next, we explored the biological functions of the 17 osteoporosis-associated miRNAs with DIANA Tools. The 38 KEGG pathways were significantly enriched such as ErbB signaling pathway, Wnt signaling pathway, AMPK signaling pathway, TGF-beta signaling pathway, and MAPK signaling pathway (Supplementary Table 1). The 222 osteoporosis-associated genes were also subjected to KEGG pathway analysis with “KEGG” package in R. And 14 KEGG pathways were significantly enriched such as cysteine and methionine metabolism, Salmonella infection, focal adhesion, gap junction, pathogenic Escherichia coli infection, glycerolipid metabolism, PI3K-Akt signaling pathway, and p53 signaling pathway (Figure 3(a)).

Importantly, 3 shared KEGG pathways were obtained including focal adhesion, PI3K-Akt signaling pathway, and gap junction. And we identified 14 miRNAs and 13 genes in the 3 shared pathways (Tables 3 and 4). Then, hub miRNAs and genes were selected according to the following criterions: (1) there was a negative regulatory relationship between miRNAs and corresponding genes; (2) the gene was the target of the miRNA predicted by miRWalk; and (3) the miRNA and corresponding gene were in the same signaling pathway. Then, we detected 6 hub miRNAs (hsa-miR-18b-3p, hsa-miR-361-3p, hsa-miR-484, hsa-miR-519e-5p, hsa-miR-940, and hsa-miR-1275) and 6 hub genes (THBS1, IFNAR2, ARHGAP5, TUBB2B, FLNC, and NTF3). To explore the regulation relationship between miRNAs and genes, we constructed a miRNA-gene interaction network (Figure 3(b)). Hsa-miR-1275 and hsa-miR-940 were the core regulators as they targeted 5 genes, respectively. And IFNAR2 and ARHGAP5 were the core node genes as they were targeted by 6 miRNAs. Besides, the 6 hub genes were all upregulated, and 6 hub miRNAs were downregulated in osteoporosis (Figures 3(c) and 3(d), *P* < 0.05).

**Figure 4:**
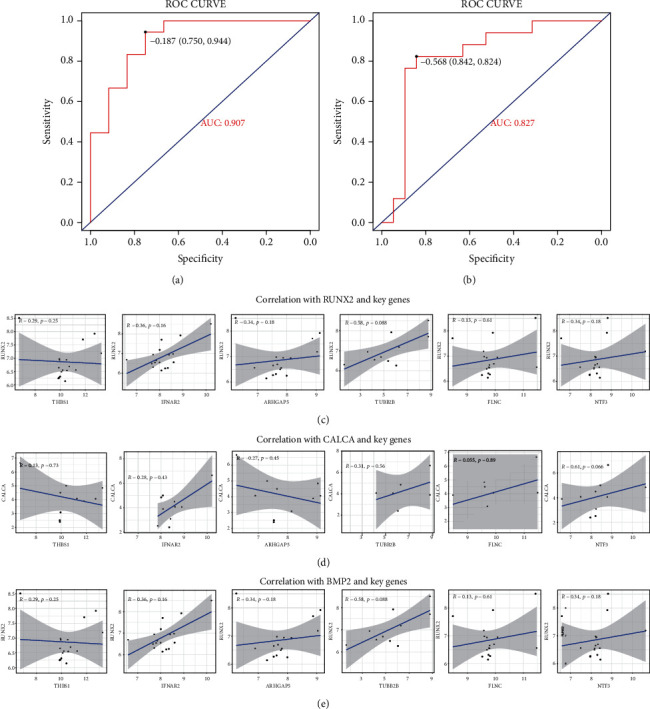
The ROC curves of hub miRNAs and core genes were plotted and correlations with these key genes and vital osteoporosis-associated factors were assessed. (a and b) The AUCs of ROC curves about hub miRNAs and core genes were calculated. (c–e) Correlation coefficients with these key genes and vital osteoporosis-associated factors including RUNX2 (c), CALCA (d), and BMP2 (e) were exhibited.

To determine the predictive performance of hub miRNAs and genes, the AUCs of ROC curves about hub miRNAs and core genes were calculated as 0.907 and 0.827 (Figures 4(a) and 4(b)), suggesting that these key miRNAs and genes had predictive actions for osteoporosis. In addition, correlation coefficients with these key genes and vital osteoporosis-associated factors including RUNX2, CALCA, and BMP2 were presented to be a trend of correlation with osteoporosis (Figures 4(c)–4(e)); complementally, these correlations coefficients were not significant, caused by insufficient sample size, the presence of missing values, and the existence of outliers. Overall, these data show a demonstrative effect of these key miRNAs and genes for osteoporosis.

### 3.4. Screening of Potential Drugs

**Figure 5:**
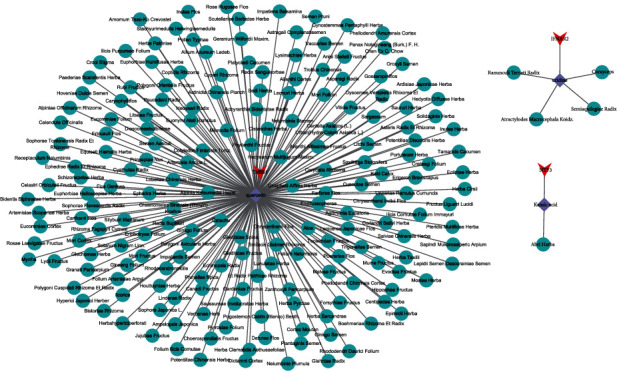
A target-compound-herb network was constructed to visualize their relationship among the 3 genes (NTF3, IFNAR2, and THBS1), 3 compounds (kainic acid, uridine, and quercetin), and 192 herbs.

**Figure 6:**
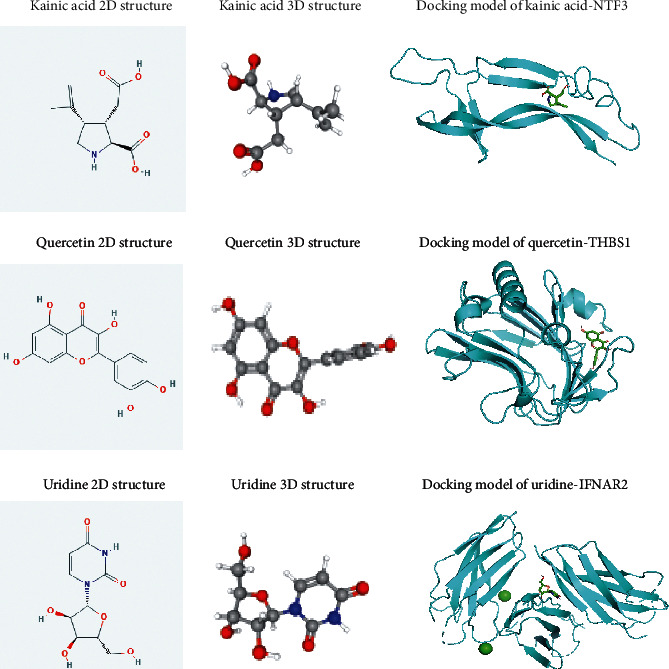
Molecular docking models were constructed with the 3 compounds (kainic acid, uridine, and quercetin) and 3 targets (NTF3, IFNAR2, and THBS1).

Next, we screened potential drugs for osteoporosis in TCMSP (Traditional Chinese Medicine Systems Pharmacology Database and Analysis Platform) based on the 6 hub genes. And 3 genes (NTF3, IFNAR2, and THBS1) were targeted by 3 natural compounds (kainic acid, uridine, and quercetin), which were the active ingredients of 192 herbs. Then, we developed a target-compound-herb network to visualize the relationship among the 3 genes, 3 compounds, and 192 herbs (Figure 5). For example, kainic acid, the active ingredient in herb Abri Herba, could affect osteoporosis by targeting NTF3; uridine, the active ingredient in herbs Atractylodes, Cordyceps, and Semiaquilegia, could affect osteoporosis by targeting IFNAR2; quercetin, the active ingredient in herbs Ardisia japonica Herba, Folium Artemisiae Argyi, Anisi stellati fructus, and Ginkgo Semen could affect osteoporosis by targeting THBS1. To investigate the relationship between compounds and the targets, molecular docking was performed.  Figure 6 displays the 3 best molecular docking models with minimum free energy. The 3 compounds were, respectively, buried in the pockets of the 3 proteins, suggesting that the 3 compounds could be potential drugs of osteoporosis targeting the 3 genes.

## 4. Discussion

WGCNA is widely used to detect the coexpressed genes correlated with certain phenotype by developing a correlation network. Currently, accumulating studies have performed WGCNA to investigate the interactions between molecules in osteoporosis. For example, Qian et al. identified PPWD1 as a potential biomarker for osteoporosis [14]. Using WGCNA, Hu et al. discovered 7 genes that affected the progression of osteoporosis [15]. In this study, we detected osteoporosis-associated miRNAs and genes using WGCNA and differential expression analyses. We identified 5 modules for miRNAs, of which the turquoise module was the most strongly correlated with osteoporosis. For genes, 9 modules were grouped, and the darkolivegreen module was the most relevant to osteoporosis. Subsequently, 17 miRNAs and 222 genes were obtained associated with osteoporosis after comparing WGCNA and differentially expression results. For miRNAs, KEGG pathway analysis indicated that the 17 miRNAs were mainly enriched in 38 signaling pathways. Meanwhile, the 222 genes were mainly enriched in 14 pathways. And 3 shared KEGG pathways including focal adhesion, PI3K-Akt signaling pathway, and gap junction were discovered. Multiple studies have demonstrated the roles of the 3 signaling pathways in osteoporosis. Focal adhesions (FAs) are macromolecules that mediate the interactions between cells and extracellular matrix [16]. Multiple studies have reported that FAs participated in cell differentiation, mobility, and angiogenesis. H. Xie et al. demonstrated that FA signaling ameliorates the progression of osteoporosis by promoting the formation of H-type vessels in bone mesenchymal stem cells [16]. Besides, Hu et al. displayed that enhanced vessels in bones were positively associated with the level of FA kinases [17]. Phosphoinositide 3-kinase- (PI3K-) protein kinase B (AKT) signaling pathway, involved in cell survival, apoptosis, and growth, has been indicated to play critical roles in regulating osteoporosis development [18]. For example, the activation of PI3K-Akt signaling pathway elevated the expression of osteogenic differentiation genes such as ALP and BMP2 to promote the growth and differentiation of osteoblast [19]. However, the inactivation of PI3K-Akt signaling attenuated bone resorption of osteoclasts [20]. Gap junction proteins were mainly identified in osteocytes, osteoblasts, and osteoclasts. Many investigations have indicated that gap junction proteins are critical to the conservation of bone structure and formation. Gap junctions were decreased in osteocytes in mouse osteoporosis model [21]. Connexin 43, a gap junction protein, is essential for the function of osteocyte and reduced in high glucose-induced osteoporosis [22]. These investigations all indicated the inhibitory roles of these signaling pathways in osteoporosis progression.

After construction the miRNA-gene interaction network, we identified 6 hub miRNAs (hsa-miR-18b-3p, hsa-miR-361-3p, hsa-miR-484, hsa-miR-519e-5p, hsa-miR-940, and hsa-miR-1275) and 6 hub genes (THBS1, IFNAR2, ARHGAP5, TUBB2B, FLNC, and NTF3) for osteoporosis. Consistently, the functions of some of these miRNAs and genes in osteoporosis have been investigated. Z. Wang et al. exhibited that miR-361 has been upregulated in osteoporosis patients [23]. MiR-484 has been discovered to positively associate with bone mineral density in in femoral bone [24]. Hashimoto et al. demonstrated that miR-940 could induce osteogenic differentiation by targeting ARHGAP1 and FAM134A [25]. The inhibition of thrombospondin-1 (THBS1) could suppress the formation of osteoclast and has reported to be involved in osteoclastogenesis [26]. Dexamethasone could mediate the osteoblast differentiation by increasing the level of TUBB2B [27]. And in our study, for the first time, the intimate association of the other 3 miRNAs (hsa-miR-18b-3p and hsa-miR-519e-5p) and 4 genes (IFNAR2, ARHGAP5, FLNC, and NTF3) with osteoporosis was discovered. However, the biological functions of the molecules in osteoporosis await to be investigated.

Currently, estrogen and bisphosphonate have been widely used in the treatment of osteoporosis. However, their side effects greatly limited their application in osteoporosis. For example, the use of estrogen elevated the incidence rate of breast cancer and heart failure [28]. Meanwhile, bisphosphonate compounds led to the irritation in gastrointestinal tract [28]. Therefore, given rare adverse effects, TCHMs, including iridoid glycosides, saponins, and flavonoids, are increasingly applied in osteoporosis. In this study, we built a target-compound-herb network using TCMSP and screened out 3 natural compounds (kainic acid, uridine, and quercetin) targeting 3 genes (NTF3, IFNAR2, and THBS1). Subsequently, the corresponding molecular docking models were constructed. For the 3 natural compounds, there were several studies related to the application of quercetin, a natural flavonoid, in osteoporosis. For example, quercetin has been reported to alleviate the progression of osteoporosis by enhancing osteogenic differentiation via AMPK signaling pathway [29]. For uridine, Li et al. demonstrated that uridine triphosphate, a kind of nucleotides, could inhibit osteogenic differentiation and promote adipogenic differentiation [30]. There was no study about therapeutic potential of kainic acid and uridine in osteoporosis. Therefore, further investigations are needed for elucidate the role of the two compounds in the treatment for osteoporosis.

## 5. Conclusion

In conclusion, our study detected 6 hub miRNAs and 6 hub genes associated with osteoporosis using comprehensive bioinformatics analysis including WCGNA, differential expression, and KEGG pathway analyses. Then, the miRNA-gene interaction network was constructed. Predictive performances of hub miRNAs and genes were evaluated, and correlations with key genes and vital osteoporosis-associated factors were determined. And 3 natural compounds (kainic acid, uridine, and quercetin) were screened out based on the hub genes. Our study sheds light on the pathogenesis of osteoporosis and provides promising therapeutic targets and drugs for osteoporosis. Some limitations of this study including correlations between targets and osteoporosis-related factors and specific mechanism of target-compound will be explored in the future work by larger samples and silencing/overexpressing the targets combined with compound administration.

## Figures and Tables

**Table 1:** Characteristics of the individual studies incorporating into the analysis of miRNA sequence.

GEO ID	Country	Year	Osteoporosis to healthy ratio	Sample organism
GSE74209	Spain	2015	6 : 6	Homo sapiens
GSE93883	Hong Kong	2020	12 : 6	Homo sapiens

**Table 2:** Characteristics of the individual studies incorporating into the analysis of mRNA sequence.

GEO ID	Country	Year	Osteoporosis to healthy ratio	Sample organism
GSE7158	China	2008	12 : 14	Homo sapiens
GSE35956	Germany	2012	5 : 5	Homo sapiens

**Table 3:** Genes identified in 3 KEGG pathways including focal adhesion, PI3K-Akt signaling pathway, and gap junction.

mRNA	log_2_FC	Adj. *P* val
TUBB2B	-1.804966996	0.044326216
THBS1	-1.457613401	0.01412781
EIF4B	-0.964608275	0.003633295
NTF3	-0.894636294	0.014447327
IFNAR2	-0.70778604	0.022075193
FLNC	-0.697736251	0.028265813
ARHGAP5	-0.663850508	0.026211769
TUBB6	0.625145836	0.045587962
PDGFRA	0.753914676	0.02396403
DOCK1	0.791003891	0.020771285
CHAD	1.029667417	0.047667364
LPAR1	1.303788899	0.013978794
ITGB8	1.726835004	0.005026655

**Table 4:** MiRNAs identified in 3 KEGG pathways including Focal adhesion, PI3K-Akt signaling pathway and Gap junction.

miRNA	log2FC	Adj. *P* val
hsa-miR-5701	-1.638326569	0.004828665
hsa-miR-4258	0.952752859	0.006681939
hsa-miR-4667-3p	0.847227866	0.008128556
hsa-miR-4640-3p	0.882432241	0.008128556
hsa-miR-361-3p	1.003944592	0.009802755
hsa-miR-18b-3p	0.85483541	0.012724624
hsa-miR-4687-3p	0.871408616	0.019689352
hsa-miR-940	0.621601199	0.025433579
hsa-miR-1275	0.887163084	0.028126477
hsa-miR-99a-5p	0.760586238	0.041045519
hsa-miR-3127-3p	0.569654097	0.043355097
hsa-miR-519e-5p	0.796422805	0.045674943
hsa-miR-3162-3p	0.574105156	0.048288935
hsa-miR-484	0.587399648	0.049103596

## Data Availability

The data that support the findings of this study are available in GEO at (https://www.ncbi.nlm.nih.gov/geo/).

## Supplementary Materials

Supplementary Materials Supplementary Figure 1 Analysis of scale-free network topology for various soft thresholding powers. (A) No outliers were detected in the 30 samples. (B) The scale-free fit index and the mean connectivity for various soft thresholding powers were analyzed, and the scale-free topology was checked when *β* = 3. Supplementary Table 1: the 38 KEGG pathways enrichment for 17 osteoporosis-associated miRNAs with DIANA Tools.
