# Supplementary material for: miRNA-Gene Interaction Network Construction Strategy to Discern Promising Traditional Chinese Medicine against Osteoporosis
Source: Biomed Res Int. 2022 Jun 15;2022:9093614. doi: 10.1155/2022/9093614 (PMC9217536; doi:10.1155/2022/9093614)

Supplementary Table 1. The 38 KEGG pathways enrichment for 17 osteoporosis-associated miRNAs with DIANA Tools.

**KEGG pathway**	**p-value**	**#genes**	**#miRNAs**
Hippo signaling pathway	5.91E-06	57	12
ErbB signaling pathway	0.000213425	39	11
Sulfur metabolism	0.001881816	5	4
Glioma	0.001881816	26	10
Morphine addiction	0.001881816	38	11
Amphetamine addiction	0.001881816	26	12
Wnt signaling pathway	0.001881816	48	13
Pathways in cancer	0.001881816	122	14
Proteoglycans in cancer	0.001881816	70	14
Thyroid hormone signaling pathway	0.001989269	41	11
GABAergic synapse	0.002041831	32	10
Adherens junction	0.002106311	27	8
Glycosaminoglycan biosynthesis - chondroitin sulfate / dermatan sulfate	0.002838718	7	6
Prostate cancer	0.00322482	35	11
Signaling pathways regulating pluripotency of stem cells	0.003401623	49	13
AMPK signaling pathway	0.004374064	43	13
Oxytocin signaling pathway	0.004900824	59	10
Adrenergic signaling in cardiomyocytes	0.005511073	49	12
TGF-beta signaling pathway	0.00752451	29	9
Dilated cardiomyopathy	0.008399411	35	11
Chronic myeloid leukemia	0.016309114	27	9
Focal adhesion	0.016309114	69	11
Rap1 signaling pathway	0.016309114	68	15
Dorso-ventral axis formation	0.01688309	14	6
Arrhythmogenic right ventricular cardiomyopathy (ARVC)	0.01688309	30	9
Bacterial invasion of epithelial cells	0.016982288	32	9
Basal cell carcinoma	0.018458213	24	11
MAPK signaling pathway	0.019361511	82	14
Hypertrophic cardiomyopathy (HCM)	0.022835931	31	9
cAMP signaling pathway	0.022888002	65	13
Ras signaling pathway	0.022888002	71	14
Melanogenesis	0.023482663	35	13
Gap junction	0.02819419	26	10
Steroid biosynthesis	0.031068373	7	5
Colorectal cancer	0.032300498	22	8
PI3K-Akt signaling pathway	0.042675225	100	14

Axon guidance	0.047095019	45	10
mTOR signaling pathway	0.047892665	23	9

Supplementary Figure 1. Analysis of scale-free network topology for various soft-thresholding powers. A, No outliers were detected in the 30 samples. B, The scale-free fit index and the mean connectivity for various soft-thresholding powers were analyzed and the scale-free topology was checked when β = 3.

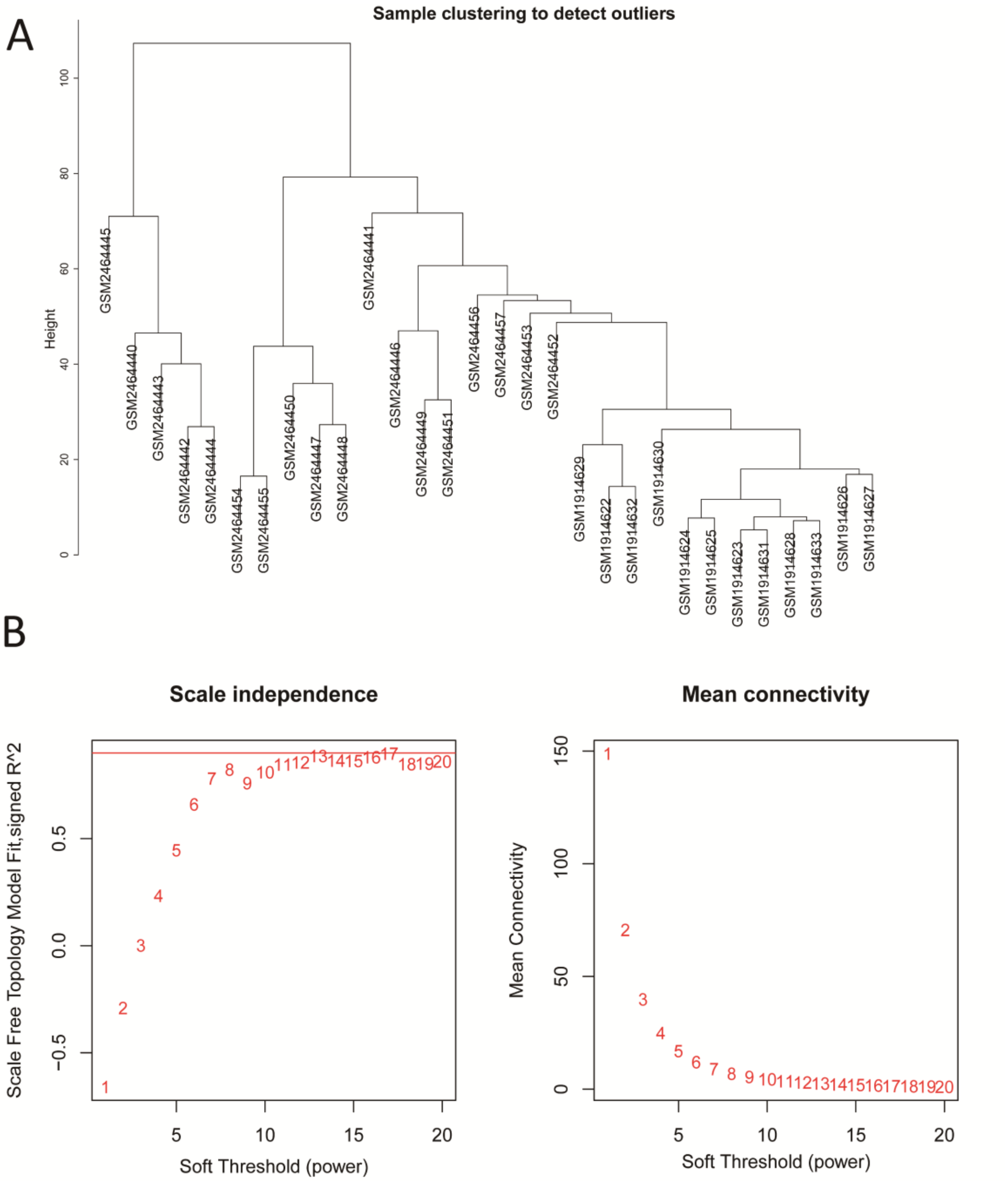

Supplement: Supplementary Materials — Supplementary Figure 1 Analysis of scale-free network topology for various soft thresholding powers. (A) No outliers were detected in the 30 samples. (B) The scale-free fit index and the mean connectivity for various soft thresholding powers were analyzed, and the scale-free topology was checked when β = 3. Supplementary Table 1: the 38 KEGG pathways enrichment for 17 osteoporosis-associated miRNAs with DIANA Tools. [file 9093614.f1.pdf]
